# Allergic Potential and Immunotoxicity Induced by Topical Application of 1-Chloro-4-(Trifluoromethyl)Benzene (PCBTF) in a Murine Model

**DOI:** 10.1155/2011/238513

**Published:** 2011-05-14

**Authors:** Jennifer Franko, Laurel G. Jackson, B. Jean Meade, Stacey E. Anderson

**Affiliations:** Health Effects Laboratory Division, National Institute for Occupational Safety and Health (NIOSH), 1095 Willowdale Drive, Morgantown, WV 26505-2888, USA

## Abstract

The purpose of the studies in this paper was to evaluate the allergic potential, immunotoxicity, and irritancy of the occupationally relevant chemical, 1-chloro-4-(trifluoromethyl)benzene, also known as parachlorobenzotrifluoride (PCBTF), following dermal exposure in a murine model. Evaluation of the sensitization potential, conducted using the local lymph node assay (LLNA) at concentrations ranging from 50% to 100%, identified a dose-dependent increase in lymphocyte proliferation with a calculated EC3 value of 53.1%. While no elevations in total or specific IgE were observed after exposure to any concentration of the chemical, significant increases in IFN-*γ* protein production by stimulated draining lymphoid cells were observed, indicating a T-cell-mediated response. Dermal exposure to PCBTF was not found to alter the immune response to a T-cell-dependant antigen. These results demonstrate that PCBTF has the potential to induce allergic sensitization following dermal exposure and based on LLNA results would be classified as a weak sensitizer.

## 1. Introduction

1-Chloro-4-(trifluoromethyl)benzene, a fluorinated toluene, also known as parachlorobenzotrifluoride (PCBTF), is a chemical used as an intermediate in a wide range of organic reactions for the synthesis of dyes, pharmaceuticals, pesticides, insecticides and herbicides [1]. It is primarily used as a solvent in commercial surface finishes, such as vapor degreasing, precision wipe cleaning, cold cleaning and electronics cleaning, and is manufactured in both pure and blended formulations based upon specific cleaning requirements [2, 3]. It is also used as an ink solvent in the printing industry and is a component (5–12%) of low volatile organic compound (VOC) compliant polyurethane finishes. PCBTF is not considered to be an air toxin or ozone depleter. It has therefore recently been considered exempt from VOC regulations [4], which has led to an increase in its use as a replacement for other solvents previously used in the manufacture of a variety of commercially-available paints, inks, and other products and finishes (Oxsol 100, Occidental Chemical Co.) [1].

PCBTF was recently nominated by the National Toxicology Program (NTP) for toxicological characterization due to its unknown chronic toxicity profile and changes in its industrial and consumer use [1]. In addition, its improper use or disposal may lead to an increase in public exposure outside of the occupational context. There are currently no Occupational Safety & Health Administration (OSHA), National Institute for Occupational Safety and Health (NIOSH), or American Conference of Governmental Industrial Hygienists (ACGIH) limits regulating PCBTF exposure [1]. 

Although the health effects of PCBTF have not been thoroughly tested, epidemiological studies in workers have reported increases in respiratory and stomach cancers [5]. Animal studies investigating the health effects of PCBTF exposure are limited. In a 13-week inhalational study in rats, no changes were identified in any measured clinical chemistry parameter, at doses up to 252 ppm, and no adverse observations were recorded during exposures or during detailed weekly clinical evaluations [6]. Subchronic studies were negative for precancerous hematological changes and other histopathological indicators of carcinogenicity [7]. However, exposure to PCBTF did increase relative liver weights between dose groups. Subchronic inhalational and oral exposure to higher concentrations of PCBTF in rats produced clinical signs of toxicity that included salivation, tremors, altered hematological, and hepatocellular profiles [7]. These signs, however, were only noted at 1000 mg/kg/day, which is greatly outside of the range expected in a potential spill of PCBTF-containing paint products. PCBTF has low subchronic oral toxicity as well, and neither pathological nor adverse biochemical effects were found at doses up to 10 mg/kg/day, which have been described as the no-observable-effect level of PCBTF [8].

Although it is a primary route of occupational exposure, very few dermal exposure studies have been conducted on this chemical [2]. In addition, immunotoxicological studies are lacking. The recent increase in occupational use, along with the potential for dermal exposure warrants the evaluation of the immunotoxicity of PCBTF following dermal exposure.

## 2. Material and Methods

### 2.1. Test Articles and Chemicals

**Figure 1:**
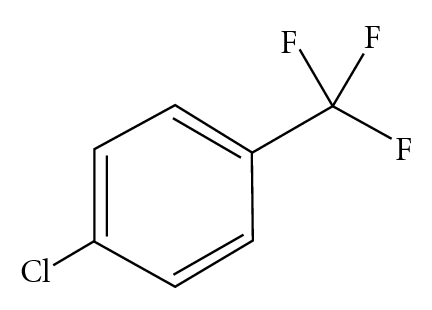
Chemical structure of PCBTF.

1-Chloro-4-(trifluoromethyl)benzene (98%) (PCBTF; Figure 1) [CAS no. 98-56-6], alpha-hexylcinnamaldehyde (HCA) [CAS no. 101-86-0], 2,4-dinitrofluorobenzene (DNFB) [CAS no. 70-34-8], toluene 2,4-diisocyanate (TDI, CAS 584-84-9) and cyclophosphamide [CAS no. 50-18-0] were all purchased from Aldrich Chemical Company, Inc. (Milwaukee, Wis).

### 2.2. Species Selection

Female BALB/c and B6C3F1 mice were used in these studies. BALB/c mice have a Th2 bias and are commonly used to evaluate potential IgE-mediated sensitization. They were therefore used in the hypersensitivity studies [9, 10]. B6C3F1 mice are the strain of choice for immunotoxicity studies and were used to evaluate the IgM response to SRBC [11]. The mice were purchased from Taconic (Germantown, NY) at 6–8 weeks of age. Upon arrival, the animals were allowed to acclimate for a minimum of 5 days. Each shipment of animals was randomly assigned to a treatment group, weighed, and individually identified via tail marking using a permanent marker. A preliminary analysis of variance on body weights was performed to ensure a homogeneous distribution of animals across treatment groups. The animals were housed at a maximum of 5 per cage in ventilated plastic shoebox cages with hardwood chip bedding. NIH-31 modified 6% irradiated rodent diet (Harlan Teklad), and tap water was provided from water bottles,* ad libitum*. The temperature in the animal facility was maintained between 68 and 72°F and the relative humidity between 36 and 57%. The light/dark cycle was maintained on 12-hour intervals. All animal experiments were performed in the AAALAC accredited NIOSH animal facility in accordance with an animal protocol approved by the Institutional Animal Care and Use Committee.

### 2.3. Concentration Range Finding Studies

Concentration range finding studies were performed to select the concentrations of PCBTF to be used for dermal exposures. BALB/c mice were exposed topically to acetone vehicle or increasing concentrations of PCBTF up to 100% in acetone on the dorsal surface of each ear (25 *μ*L per ear) for three consecutive days. Animals were allowed to rest for 2 days following the last exposure and then weighed and examined for signs of toxicity, such as loss of body weight, fatigue/lack of activity, and ungroomed fur. The maximum concentration selected for the subsequent studies was based on limits of toxicity.

### 2.4. Combined Local Lymph Node and Irritancy Assay

To determine the irritancy and sensitization potential of PCBTF, a combined local lymph node assay (LLNA) was conducted. PCBTF dosing concentrations (50–100%) and vehicle (acetone) were selected based on solubility and preliminary concentration range finding studies. The LLNA was performed according to the method described in the ICCVAM Peer Review Panel report (1999) with minor modifications [12]. Briefly, mice (5 per group) were topically treated with acetone vehicle, increasing concentrations of PCBTF, or positive control (30% alpha-hexylcinnamaldehyde; HCA) on the dorsal surface of each ear (25 *μ*L per ear) once a day for three consecutive days. 2,4-dinitrofluorobenzene (DNFB) was used as a positive control for irritancy. Irritancy measurements were performed as previously described [13]. The thickness of the right and left ear pinnae of each mouse was measured using a modified engineer's micrometer (Mitutoyo Co.) before the first chemical administration and 24 hours following the final exposure. The mean percentage of ear swelling was calculated based on the following equation: [(mean postchallenge ear thickness − mean prechallenge ear thickness)/mean prechallenge thickness] × 100. Animals were allowed to rest for 2 days following the last exposure. On day 6 mice were injected intravenously, via the lateral tail vein, with 20 *μ*Ci ^3^H-thymidine (Dupont NEN; specific activity 2 Ci/mmol). Five hours after ^3^H-thymidine injection, animals were euthanized via CO_2_ inhalation, and the left and right superficial parotid draining lymph nodes (DLNs), located at the bifurcation of the jugular vein, were excised and pooled for each animal. Single cell suspensions were made and incubated overnight in 5% trichloroacetic acid (TCA), and samples were counted using a Packard Tri-Carb 2500TR liquid scintillation analyzer (Perkin Elmer). Stimulation indices (SI) were calculated by dividing the mean disintegrations per minute (DPM) per test group by the mean DPM for the vehicle control group. EC3 values (concentration of chemical required to induce a 3-fold increase over the vehicle control) were calculated based on the equations from Basketter et al. [14].

### 2.5. Phenotypic Analysis of Lymphocytes

Lymphocyte phenotypes were analyzed using flow cytometry as described by Manetz and Meade [15]. For the phenotypic analysis, mice were topically exposed to acetone or increasing concentrations of PCBTF (up to 100%) on the dorsal surface of each ear (25 *μ*L per ear) once a day for four consecutive days. Animals were allowed to rest for 6 days after the final treatment and then euthanized on day 10 by CO_2_ inhalation. Animals were weighed and examined for gross pathology at the end of the experiment. The following organs were removed, cleaned of connective tissue and weighed: liver, spleen, kidneys, and thymus. DLNs (two nodes/animal/tube) and spleens were also collected separately in 3 mL PBS and were dissociated using the frosted ends of two microscope slides. Cell counts were performed using a Coulter Counter (Z2 model, Beckman Coulter), and 1 × 10^6^ cells per sample were added to the wells of a 96-well plate. Cells were washed using staining buffer (1% bovine serum albumin/0.1% sodium azide in PBS) and then incubated with Fc block (clone 2.4G2). For IgE+/B220+ analysis, the cells were incubated with anti-CD45RA/B220 (PE, clone RA3-6B2) and anti-IgE antibodies (FITC, clone R-35-72) or appropriate isotype control diluted in staining buffer. For analysis of T-cell subsets, cells were incubated with anti-mouse CD3e antibody (APC, clone 145-2C11), anti-mouse CD4 antibody (FITC, clone RM4-5), and anti-mouse CD8a antibody (PE, clone 53-6.7) or the appropriate isotype controls diluted 1 : 100 in staining buffer. All antibodies and isotype controls were purchased from BD Pharmingen. Cells were then washed and incubated with propidium iodide (PI). After a final wash, cells were resuspended in staining buffer and analyzed with a Becton Dickinson FACSCalibur flow cytometer using a PI viability gate.

### 2.6. Total Serum IgE

For analysis of total IgE, PCBTF was tested at concentrations up to 100%. Mice were treated with acetone, increasing concentrations of PCBTF, or, as a positive control, 1.5% TDI on the dorsal surface of each ear (25 *μ*L per ear) once a day for four consecutive days. Animals were allowed to rest for 6 days after the final treatment and were euthanized on day 10 by CO_2_ inhalation. Following euthanasia, blood samples were collected via cardiac puncture. Sera were separated by centrifugation (10 min at 10,000 × g) and frozen at −20°C for next day analysis of IgE by ELISA. A standard colorimetric sandwich ELISA was performed as previously described [16]. All antibodies and isotype controls were purchased from BD Pharmingen. In brief, 96-well flat bottom plates (Dynatech Immulon-2) were coated with purified monoclonal rat anti-mouse IgE antibody (clone R35-72; 2 *μ*g/mL, diluted in 0.05 M carbonate-bicarbonate buffer, pH 9.6), sealed with plate sealers, and incubated overnight at 4°C. The plates were washed 3 times with PBS/Tween 20 and then blocked for 1 hour with diluent (2% fetal bovine serum (FBS; Hyclone Laboratories, Inc., Logan, Utah) and 0.05% sodium azide) at room temperature. Serum samples were diluted 1 : 10 in diluent and IgE control standards (mouse IgE anti-TNP, clone C38-2) were prepared (highest concentration: 500 ng/mL). The diluted serum samples and IgE control standards were then serially diluted (1 : 2) through 8 wells, added to the coated plates in a 100 *μ*L volume and incubated at room temperature for 1 hour. The plates were washed 3 times with PBS/0.05% Tween 20. Biotin-conjugated rat anti-mouse IgE (clone R35-92; 2 *μ*g/mL) was added in a 100 *μ*L volume and plates were incubated at room temperature for 1 hour. The plates were then washed 3 times with PBS/0.05% Tween 20. Streptavidin-alkaline phosphatase was added (100 *μ*L of a 1 : 400 in diluent) and plates were incubated for 1 hour at room temperature. P-Nitrophenyl phosphate (Sigma) was used as the alkaline phosphatase substrate and added to the plates in a 100 *μ*L volume. The plates were allowed to develop for up to 30 minutes at room temperature or until the optical density (OD) reading of the highest standard reached 3.0. Absorbance was determined using a Spectramax Vmax plate reader (Molecular Devices) at 405–605 nm. Data analysis was performed using the IBM Softmax Pro 3.1 (Molecular Devices), and the IgE concentrations for each sample were interpolated from a standard curve using multipoint analysis.

### 2.7. Analysis of Cytokine Production by Draining Lymph Node Cells

To determine cytokine protein production by lymphocytes, DLNs of mice used for the analysis of total IgE were collected (two nodes/animal/tube) in 2 mL PBS and dissociated using the frosted ends of two microscope slides. Cell counts were performed using a Coulter Counter (Z1 model, Beckman Coulter), and cells were adjusted to 1 × 10^6^ cells/mL using sterile RPMI media containing 10% FBS. Cells were added to a 48-well plate in a 500 *μ*L volume, stimulated with *α*-CD3 and *α*-CD28 (2 *μ*g/mL of each; BD Pharmingen) and incubated for 24 hours at 37°C and 5% CO_2_. Supernatants were analyzed for IL-4 and IFN-*γ* production using an OptEIA ELISA kit purchased from BD Biosciences according to the manufacturer's instructions. Supernatants collected from each culture (2 stimulated and 2 unstimulated for each mouse) were added to the plates in triplicate along with serial dilutions of the standards. Plates were read at 450 nm [OD values for standards ranging from 0.77–1.93] using a SpectraMax M2 spectrophotometer (Molecular Devices). Cytokine concentration was extrapolated from the standard curve. The final data are expressed as the mean value generated when the concentration identified for the unstimulated cultures was subtracted from the value generated from the stimulated cultures for each mouse.

### 2.8. *In Vivo* IgM Response to the T-Cell-Dependent Antigen, SRBC

The primary IgM response to sheep red blood cells (SRBC) was enumerated using a modified hemolytic plaque assay of Jerne and Nordin [17]. B6C3F1 mice were dermally exposed to PCBTF (6–100%) for 14 days (25 *μ*L/ear). Four days prior to euthanasia (day 11), the mice were immunized with 7.5 × 10^7^ SRBC by intravenous injection in a 200 *μ*L volume. All SRBC for these studies were drawn from a single donor animal (Lampire Laboratories, Pipersville, Penn). On the day of sacrifice, mice were euthanized by CO_2_ asphyxiation, body and organ weights were recorded and spleens were collected in 3 mL of Hanks Balanced Salt Solution (HBSS). Single cell suspensions of the spleens from individual animals were prepared in HBSS by disrupting the spleen between the frosted ends of microscopic slides. To identify the total number of spleen cells, 20 *μ*L of cells were added to 10 mL of isoton buffer (1 : 500) and two drops of Zap-o-globin were added to lyse red blood cells. Cells were then counted using a Coulter counter. 1 : 30 and 1 : 120 dilutions of spleen cells were made. One hundred *μ*L of the dilutions were added to a test tube containing a 0.5 mL warm agar/dextran mixture (0.5% Bacto-Agar, DIFCO; and 0.05% DEAE dextran, Sigma), 25 *μ*L of 1 : 1 ratio of SRBC suspension, and 25 *μ*L of 1 : 4 dilution (1 mL lyophilized) guinea pig complement (Cedarlane Labs). Each sample was vortexed, poured into a petri dish, covered with a microscope coverslip, and incubated for 3 hours at 37°C. The plaques (representing antibody forming B-lymphocytes) were viewed and quantified after this incubation. Results were expressed as specific activity (IgM PFC per 10^6^ spleen cells) and total activity (IgM PFC per spleen).

### 2.9. Statistical Analysis

For analysis of the data generated from the described animal studies, the data were first tested for homogeneity using the Bartlett's Chi Square test. If homogeneous, a one-way analysis of variance (ANOVA) was conducted. If the ANOVA showed significance at *P* < .05 or less, the Dunnett's Multiple Range *t* test was used to compare treatment groups with the control group. Linear trend analysis was performed to determine if PCBTF had exposure concentration-related effects for the specified endpoints. Statistical analysis was performed using Graph Pad Prism version 5.0 (San Diego, CA). Statistical significance is designated by *(*P* ≤ .05) and **(*P* ≤ .01).

## 3. Results

### 3.1. *In Vivo* Studies Identified PCBTF to Be an Allergic Sensitizer

**Figure 2:**
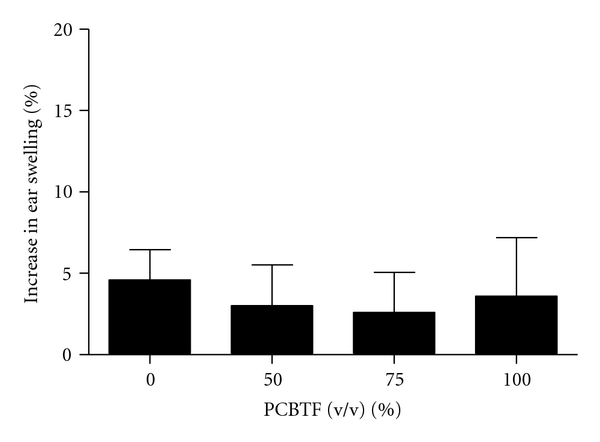
Ear swelling as a result of topical application of PCBTF. Analysis of irritation after topical application of PCBTF. Bars represent mean ± SE of 5 mice (10 ears) per group.

**Figure 3:**
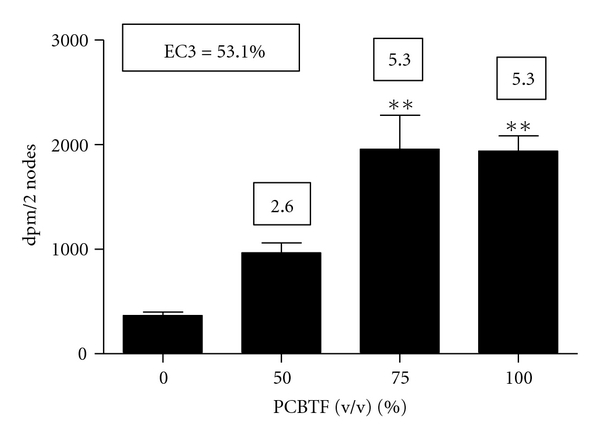
Allergic sensitization potential after dermal exposure to PCBTF. Analysis of the allergic sensitization potential of PCBTF using the LLNA. ^3^H-thymidine incorporation into draining lymph node cells of BALB/c mice following exposure to vehicle or concentration of PCBTF. Numbers in boxes appearing above the bars represent the stimulation indices for each concentration tested. Bars represent mean ± SE of 5 mice per group. Levels of statistical significance are denoted **(*P* < .01) as compared to acetone vehicle.

**Figure 4:**
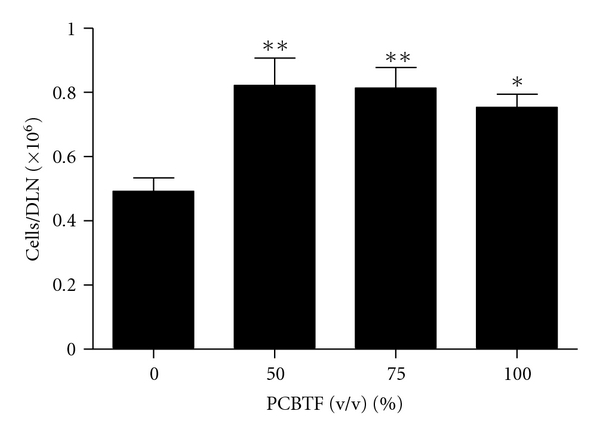
Increased cellularity of DLN after PCBTF exposure. Analysis of lymphocyte cellularity (cells/DLN) following exposure to PCBTF. Bars represent means ± SE of 5 mice per group. Levels of statistical significance are denoted as *(*P* ≤ .05) and **(*P* ≤ .01) as compared to acetone vehicle.

Dermal exposure to PCBTF was not found to be toxic at any concentration tested (data not shown). For this reason, concentrations of PCBTF up to 100% were tested in the subsequent studies. No ear swelling was observed in mice after dermal exposure to PCBTF (Figure 2), suggesting that PCBTF is nonirritating. DNFB (0.03%) was used as a positive control for irritancy studies and resulted in an average significant increase of 84% in ear swelling after application (data not shown). In the LLNA, dose-dependent (Linear Trend test; *P* < .01) increases in DLN proliferation were observed after treatment with PCBTF, with counts from the 75% and 100% PCBTF exposed animals being significantly elevated over the vehicle control animals (Figure 3). SI values were 2.6, 5.3, and 5.3 for the 50%, 75%, and 100% exposure groups, respectively. An EC3 value of 53.1% (Figure 3) was calculated. HCA (30%) was used as a positive control for these experiments and resulted in an average SI value of 24.5 (data not shown). Consistent with the LLNA results, PCBTF exposure also significantly elevated the cellularity of the DLN (Figure 4) following exposure to all concentrations.

### 3.2. Exposure to PCBTF Did Not Induce an Increase in Local or Systemic IgE Levels

**Figure 5:**
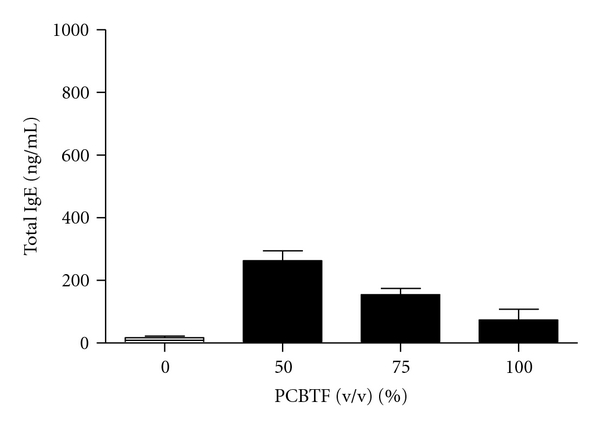
Lack of increase in serum IgE following PCBTF exposure. Analysis of total serum IgE after exposure to PCBTF. Bars represent means ± SE of 5 mice per group.

No changes in body or organ weights were observed after exposure to PCBTF for these studies (data not shown). The mechanisms of PCBTF sensitization were further investigated using phenotypic analysis of B220+ and IgE+B220+ expressing cells in the DLNs and spleen. No increases were observed in the B220+ cell or IgE+B220+ cell populations (Table 1) in the DLN or spleen. No changes in the percentage of CD4+ or CD8+ T cells in the DLN or spleen were observed after exposure to any concentration of PCBTF (data not shown). Consistent with the IgE+B220+ results, exposure to PCBTF did not elevate total serum IgE levels after exposure to any treatment groups (Figure 5). Dermal exposure to the respiratory sensitizer TDI (1.5%) significantly elevated the B220+ (19.8 ± 2.3%) cell population, IgE+B220+ (13.3 ± 2.8%) cell populations, and total IgE (1587 ± 109 ng/mL) levels (data not shown).

### 3.3. Exposure to PCBTF Increased Production of IFN-*γ*, but Not IL-4 by Stimulated DLN Cells

**Figure 6:**
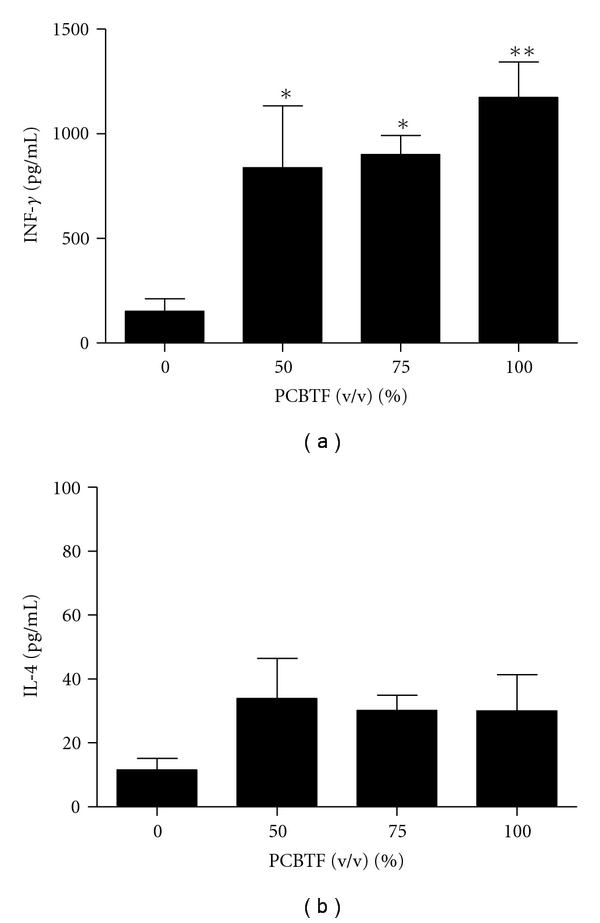
Increased IFN-*γ* protein production by DLN. Analysis of cytokine protein expression (a) IL-4 and (b) IFN-*γ* generated by stimulated DLN after dermal exposed to PCBTF. Bars represent mean fold change ± SE of 5 mice per group. Levels of statistical significance are denoted as *(*P* < .05) and **(*P* < .01) as compared to acetone vehicle.

Levels of IL-4 and IFN-*γ* cytokine production by stimulated draining lymphoid cells were analyzed to evaluate the effect of PCBTF exposure on Th1/Th2 balance. A dose-responsive (Linear Trend test; *P* < .01) increase in IFN-*γ* protein production by the DLN was observed after dermal exposure to PCBTF. Significant elevations in cytokine production were observed at PCBTF concentrations 50% and greater (Figure 6(a)). The maximum increase in INF-*γ* protein expression was calculated to be 1,174 ± 169 pg/mL. No significant alterations in IL-4 production were detected at any dose concentration (Figure 6(b)). To confirm the lack of Th2 cytokine induction following PCBTF exposure, cytokine mRNA levels were also analyzed in the DLNs of dermally exposed animals. Consistent with the protein data, no increase in IL-4 or IL-13 expression was observed (data not shown). Dermal treatment with 1.5% TDI elevated IL-4 and IL-13 mRNA expression in the DLN and significantly enhanced IL-4 protein production by stimulated draining lymphoid cells (496 ± 20 pg/mL) (data not shown). The T-cell-mediated sensitizer HCA (30%) significantly elevated IFN-*γ* expression (2,122 ± 67 pg/mL) (data not shown).

### 3.4. Dermal Exposure to PCBTF Did Not Alter the IgM Response to SRBC

**Figure 7:**
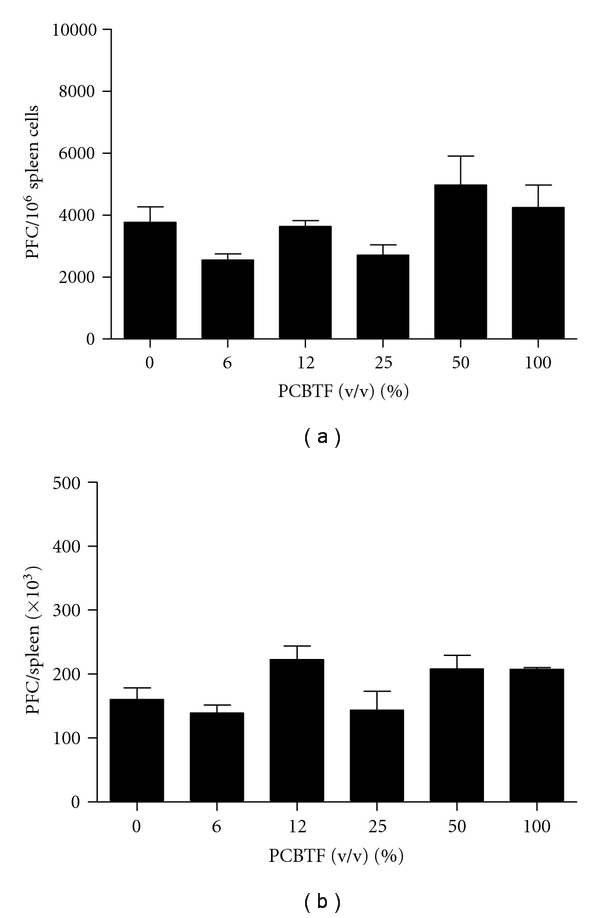
PCBTF exposure does not alter the IgM response to SRBC. Analysis of antibody producing spleen cells after a 14-day dermal exposure to PCBTF did not produce alternations in the total (a) and specific activity (b) IgM response to SRBC. Bars represent mean fold change ± SE of 5 mice per group.

To evaluate immunosuppressive potential, the murine splenic IgM response to SRBC was examined following a 14-day exposure to PCBTF. No changes in total (PFC/spleen) or specific (PFC/10^6^ cells) IgM antibody activity to SRBC were observed after exposure to any concentration of PCBTF (Figure 7). Animals exposed to the positive control, cyclophosphamide, had a significantly reduced specific spleen IgM response (67%) and total IgM response (54%) compared to the acetone control animals. No changes in body or organ weights were observed for these animals (data not shown).

## 4. Discussion

More than 13 million workers in the United States are potentially exposed to chemicals that can be absorbed through the skin. Dermal exposure to chemicals in the workplace has a great potential to affect immune function. Contact dermatitis is the second most commonly reported occupational illness responsible for up to 30% of all cases of occupational disease in industrialized nations [18]. This may result in considerable social and economic implications, including time off from work, loss of workplace productivity, reduced quality of life, and medical and worker's compensation costs, accounting for the loss of billions of dollars [19]. 

 Results from these studies suggest that PCBTF is a weak T-cell-mediated sensitizer. This was evidenced by an increase in lymphocyte proliferation and IFN-*γ* protein production by stimulated draining lymphoid cells, in the absence of elevations in markers of IgE-mediated sensitization, such as total IgE and IL-4 (mRNA and protein) production, following dermal exposure in mice. The EC3 value for PCBTF, 53.1%, falls within the working concentration for this chemical, as it is used at concentrations up to 100% as a solvent and as a chemical intermediate in the production of other chemicals. This suggests a greater predicted risk for worker sensitization. Immune suppression and other immunotoxic effects were not observed after exposure to this chemical.

Although exposure can occur through inhalation and dermal contact during its production and use [2], there are no standards in place to limit occupational exposure to PCBTF [1]. For occupational exposures, an 8-hour time-weighted average (TWA) permissible exposure level of 20 ppm has been suggested by the Kowa American Corporation, the United States chemical importation company that has renominated PCBTF for toxicological evaluation [1, 6]. In additional to occupational exposure, there is also the potential for exposure of the general public through PCBTF-containing products and PCBTF in and around ground water [20]. Detectable levels of PCBTF have been identified in workers (<1 ppm) as well as wildlife (0.17–2.0 ppm) [21, 22]. 

PCBTF is a halogenated solvent. These types of solvents are usually considered more toxic to humans and usually capable of causing greater environmental damage. Examples of other halogenated solvents include trichloroethylene, tetrachloroethylene, 1,1,1-trichloroethane, carbon tetrachloride, and dichloromethane. The majority of these solvents are classified as carcinogens and central nervous system depressants and can enter the body through respiratory or dermal exposure [23]. In addition to the effects to the central nervous system, workplace exposure to many of these solvents have been associated with toxic effects in the liver and kidney [24], as well as immune cell activation [25–31]. The effects of PCBTF exposure on immune parameters are consistent with those for other halogenated solvents, such as trichloroethylene, which has been shown to enhance activation markers on and IFN-*γ* secretion by splenic CD4+ T-cells [25–27]. Enhanced T-cell activation due to trichloroethylene exposure has been demonstrated to enhance autoimmune-related responses in both humans and mouse models [28, 29]. Due to the wide range of health effects associated with halogenated solvent exposure, occupational exposure limits have been set for these compounds, which have resulted in more stringent ventilation controls and personal protective equipment use by workers.

## 5. Conclusion

These are the first studies to indentify the sensitization potential of PCBTF following dermal exposure. Results from these studies indicate the importance of avoiding dermal contact with PCBT, due to its potential to function as a T-cell-mediated sensitizer and encourages the need to determine appropriate PPE to prevent worker exposure.

##  Disclosure

The findings and conclusions in this report are those of the authors and do not necessarily represent the views of the National Institute for Occupational Safety and Health, Centers for Disease Control and Prevention.

## Figures and Tables

**Table 1:** Phenotypic analysis after *in vivo* PCBTF treatment.

Dose group	IgE+B220+ (% lymphocyte population)		B220+ (% lymphocyte population)	
	%	Cells × 10^6^	%	Cells × 10^6^
DLN				
Acetone	1.1 ± 0.3	0.09 ± 0.02	11.5 ± 1.9	1.1 ± 0.16
PCBTF				
50%	4.5 ± 2.1	0.13 ± 0.02	14.0 ± 3.1	2.0 ± 0.53
75%	3.5 ± 0.4	0.12 ± 0.02	12.6 ± 0.9	1.8 ± 0.24
100%	2.4 ± 0.6	0.14 ± 0.01	12.0 ± 1.4	1.8 ± 0.23

Spleen				
Acetone	4.2 ± 2.4	2.1 ± 1.01	43.4 ± 4.7	2.3 ± 1.06
PCBTF				
50%	4.7 ± 2.5	2.2 ± 1.33	38.9 ± 1.8	1.7 ± 1.12
75%	1.4 ± 0.1	1.7 ± 1.58	42.6 ± 1.7	1.9 ± 0.96
100%	2.2 ± 0.9	2.1 ± 0.98	40.1 ± 1.4	2.1 ± 1.21
